# Patterns of acute ischemic stroke and intracranial hemorrhage in patients with COVID-19: Results of a retrospective multicenter neuroimaging-based study from three central European countries

**DOI:** 10.1007/s00415-023-11608-2

**Published:** 2023-02-23

**Authors:** Ulf Jensen-Kondering, Christoph J. Maurer, Hanna C. B. Brudermann, Marielle Ernst, Sam Sedaghat, Nils G. Margraf, Thomas Bahmer, Olav Jansen, Jawed Nawabi, Estelle Vogt, Laura Büttner, Eberhard Siebert, Michael Bartl, Volker Maus, Gregor Werding, Marc Schlamann, Nuran Abdullayev, Benjamin Bender, Vivien Richter, Annerose Mengel, Siri Göpel, Ansgar Berlis, Astrid Grams, Valentin Ladenhauf, Elke R. Gizewski, Philipp Kindl, Victor Schulze-Zachau, Marios Psychogios, Inke R. König, Stefan Sondermann, Sönke Wallis, Norbert Brüggemann, Peter Schramm, Alexander Neumann

**Affiliations:** 1 Department of Radiology and Neuroradiology, UKSH, Campus Kiel, Kiel, Germany; 2 Department of Neuroradiology, UKSH, Campus Lübeck, Lübeck, Germany; 3 Department of Diagnostic and Interventional Neuroradiology, University Hospital Augsburg, Augsburg, Germany; 4 Institute of Medical Biometry and Statistics (IMBS), UKSH, Campus Lübeck, Lübeck, Germany; 5 Institute of Diagnostic and Interventional Neuroradiology, University Medical Center, Göttingen, Germany; 6 Department of Radiology, University of California San Diego, San Diego, USA; 7 Department of Neurology, UKSH, Campus Kiel, Kiel, Germany; 8 Department of Internal Medicine, UKSH, Campus Kiel, Kiel, Germany; 9 Department of Radiology, Charité - Universitätsmedizin Berlin, Campus Mitte (CCM), Humboldt-Universität zu Berlin, Freie Universität Berlin, Berlin Institute of Health, Berlin, Germany; 10 Berlin Institute of Health (BIH), BIH Biomedical Innovation Academy, Berlin, Germany; 11 Institute of Neuroradiology, Charité - Universitätsmedizin Berlin, corporate member of Freie Universität Berlin and Humboldt-Universität zu Berlin, Berlin, Germany; 12 Department of Neurology, University Medical Center, Göttingen, Germany; 13 Department of Diagnostic and Interventional Neuroradiology and Nuclear Medicine, University Medical Center Knappschaftskrankenhaus, Bochum, Germany; 14 Department of Radiology, Neuroradiology Division, University of Cologne, Cologne, Germany; 15 Department of Diagnostic and Interventional Neuroradiology, University Hospital Tübingen, Tübingen, Germany; 16 Department of Neurology and Stroke, University Hospital Tübingen, Tübingen, Germany; 17 Department of Internal Medicine I, University Hospital Tübingen, Tübingen, Germany; 18 Department of Neuroradiology, Medical University of Innsbruck, Innsbruck, Austria; 19 Department of Neurology, Medical University of Innsbruck, Innsbruck, Austria; 20 Department of Neuroradiology, University Hospital Basel, Basel, Switzerland; 21 Department of Internal Medicine, UKSH, Campus Lübeck, Lübeck, Germany; 22 Department of Neurology, UKSH, Campus Lübeck, Lübeck, Germany; 23 GFO Clinics Troisdorf, Radiology and Neuroradiologie, Troisdorf, Germany

**Keywords:** COVID-19, Acute ischemic stroke, Intracranial hemorrhage, Neuroimaging

## Abstract

**Background:**

Coronavirus disease 2019 (COVID-19) is an infection which can affect the central nervous system. In this study, we sought to investigate associations between neuroimaging findings with clinical, demographic, blood and cerebrospinal fluid (CSF) parameters, pre-existing conditions and the severity of acute COVID-19.

**Materials and methods:**

Retrospective multicenter data retrieval from 10 university medical centers in Germany, Switzerland and Austria between February 2020 and September 2021. We included patients with COVID-19, acute neurological symptoms and cranial imaging. We collected demographics, neurological symptoms, COVID-19 severity, results of cranial imaging, blood and CSF parameters during the hospital stay.

**Results:**

442 patients could be included. COVID-19 severity was mild in 124 (28.1%) patients (moderate *n* = 134/30.3%, severe *n* = 43/9.7%, critical *n* = 141/31.9%). 220 patients (49.8%) presented with respiratory symptoms, 167 (37.8%) presented with neurological symptoms first. Acute ischemic stroke (AIS) was detected in 70 (15.8%), intracranial hemorrhage (IH) in 48 (10.9%) patients. Typical risk factors were associated with AIS; extracorporeal membrane oxygenation therapy and invasive ventilation with IH. No association was found between the severity of COVID-19 or blood/CSF parameters and the occurrence of AIS or IH.

**Discussion:**

AIS was the most common finding on cranial imaging. IH was more prevalent than expected but a less common finding than AIS. Patients with IH had a distinct clinical profile compared to patients with AIS. There was no association between AIS or IH and the severity of COVID-19. A considerable proportion of patients presented with neurological symptoms first. Laboratory parameters have limited value as a screening tool.

**Supplementary Information:**

The online version contains supplementary material available at 10.1007/s00415-023-11608-2.

## Introduction

The pandemic with severe acute respiratory syndrome coronavirus 2 (SARS-CoV2) which already caused more than 608 million infections and claimed more than 6.5 million lives worldwide [1] continues to be a global public health crisis with the numbers of cases surging in many places. In addition to the respiratory tract, many organs can be infected by the virus, too. Early in the pandemic, involvement of the central nervous system (CNS) has been described in a substantial proportion of patients [2]. While the association with acute ischemic stroke (AIS) is well established [3], the association with intracranial hemorrhage (IH) is less clear. The rate of CNS complications seems to be high in patients with severe disease courses and in patients hospitalized in the intensive care unit [4]. However, information on mildly and moderately affected patients is lacking.

In this study, we sought to investigate associations between neuroimaging findings with clinical parameters, severity of acute COVID-19, demographic parameters as well as pre-existing conditions and the predictive value of laboratory parameters from blood and cerebrospinal fluid (CSF).

## Materials and methods

**Fig. 1:**
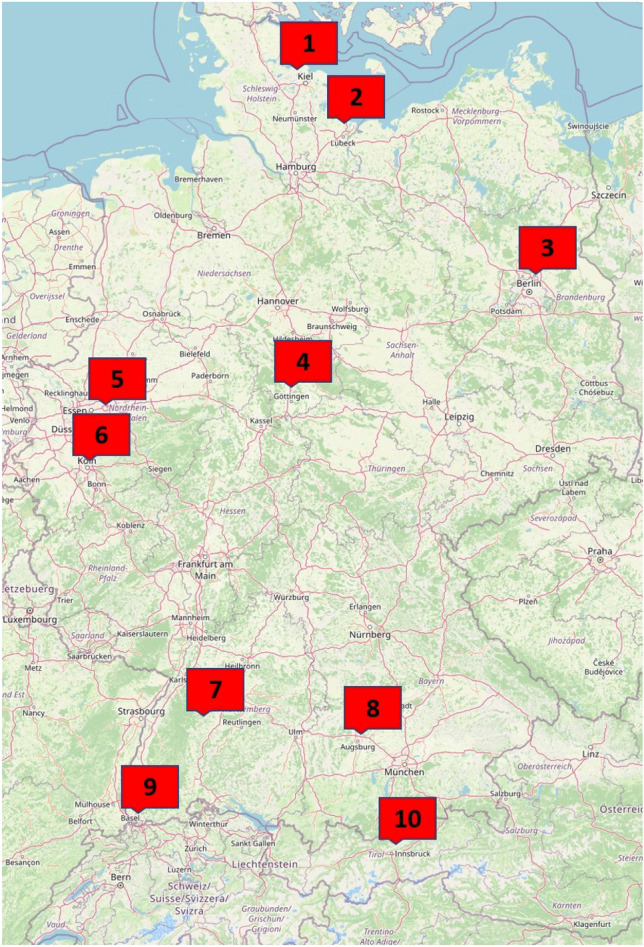
Geographic distribution of the participating centers in Germany, Switzerland and Austria. ( © OpenStreetMap, openstreetmap.org/copyright). 1: University Medical Center Schleswig–Holstein, Campus Kiel, 2: University Medical Center Schleswig–Holstein, Campus Lübeck, 3: Charité Universitätsmedizin Berlin, 4: University Medical Center Göttingen, 5: University Medical Center Knappschaftskrankenhaus Bochum, 6: University Hospital Cologne, 7: University Hospital Tübingen, 8: University Hospital Augsburg, 9: University Hospital Basel, 10: University Medical Center Innsbruck

Patients were retrospectively included from 10 university medical centers from a wide geographical range (Fig. 1) in three countries (Germany, Switzerland and Austria) between February 2020 to September 2021.

The study was approved by the Ethics Committee of the University of Kiel (D502/20 and its amendment) for central data processing and by the participating centers subject to local requirements.

We included patients who met the following inclusion criteria. (1) Acute laboratory-confirmed infection with SARS-CoV2 diagnosed by polymerase chain reaction (PCR) for SARS-CoV2 from a nasopharyngeal swab performed and processed by local standards or serological detection of SARS-CoV2 specific antibodies if a PCR was not available from the acute phase of the infection. (2) Acute symptoms of the central nervous system (3) Routine imaging of the brain using computed tomography (CT) and/or magnetic resonance imaging (MRI).

We retrospectively collected demographic information as well as clinical data related to COVID-19 and treatment, imaging data, neurological symptoms, blood and CSF parameters from the patients’ charts using a standardized template sheet. Additionally, we recorded the total number of patients hospitalized with COVID-19 at the participating centers during the study period.

### Demography

Demographic information including sex, age, pre-existing conditions and the use of antithrombotic drugs at the time of imaging was recorded.

### Clinical information

The severity of COVID-19 was assessed in a composite score according to the “Handbook COVID-19 Prevention and Treatment” [5].

Outcome was assessed as complete recovery or persistence of neurological symptoms and death during the hospital stay.

We recorded whether patients were invasively ventilated at the time of imaging and if extracorporeal membrane oxygenation (ECMO) therapy was applied. Further, the duration of invasive ventilation and ECMO therapy was recorded at the time of imaging.

### Neurological symptoms

We recorded the presence of the following neurological symptoms with acute onset: decreased level of consciousness, delirium, speech impairment (aphasia or dysarthria), visual impairment, oculomotor dysfunction, paresis and impaired sensory levels (dys-, hyp- and paresthesia). Additional data were collected concerning the presence of smell or taste dysfunction, headaches, psychiatric disorders (including impaired memory), epileptic seizures, dizziness and miscellaneous.

### Time variables

For 387 of the 442 patient (information missing in 55 patients), we recorded the dichotomized information whether neurological symptoms (“Neuro first”) or COVID-19 related respiratory and/or inflammatory symptoms (“COVID first”) occurred first.

If available, the time interval between the onset of neurological symptoms and COVID-19-related symptoms (or vice versa) and the time interval between the onset of neurological symptoms and imaging were noted.

### Imaging

For each patient, we collected the imaging results closest to the onset of neurological symptoms. We recorded acute imaging findings potentially associated with COVID-19, for example AIS including the affected vascular territory and IH including location/type of hemorrhage as well as other imaging findings potentially associated with COVID-19: venous thrombosis, acute white matter abnormalities, meningeal enhancement, olfactory nerve pathologies and miscellaneous findings potentially associated with COVID-19. Chronic imaging findings probably not associated with COVID-19 were also noted. We gathered data on imaging modality (CT or MRI), and whether contrast agent was applied. All imaging modalities at each time-point were rated at a university medical center with the diagnosis made by expert neuroradiologists.

### Statistics

We present variables as mean and standard deviation and as median and range as appropriate. To compare variables between groups we used a two-sided *t*-test for normally distributed variables and a two-sided Mann–Whitney-*U* Test for non-normally distributed variables. We applied Fisher’s exact test or a *χ*^2^-test for nominal data in 2 × 2 tables as appropriate. We performed a logistic regression model for the variables AIS and IH present or absent. In an exploratory approach, we did not perform correction for multiple testing but interpret test results descriptively. We used R version 4.0.0 for all analyses.

## Results

### Demography

442 patients (188 females, 42.5%) were included. Some of the patients have already been partially described in previous publications [6–8]. Mean age was 69 ± 16 years (range 19–99). During the study period, a total of 4115 patients with COVID-19 were treated in the participating centers, the total number of patients treated by two centers was not available. The proportion of patients included in the study (CNS symptoms and brain imaging) was 9.5% (Table 1). 91 patients had no pre-existing conditions, 110 patients had one, 98 patients had two, 93 patients had three, 39 had four and 11 patients had 5 pre-existing conditions (Table 2). Antithrombotic drugs were used in 106 patients (24.0%) for various reasons but this information was not systematically obtained.

**Table 1:** Patients included per participating center

Center	Patients included in this study	Total number of hospitalized patients
University Medical Center Schleswig–Holstein, Campus Kiel	51	372
University Medical Center Schleswig–Holstein, Campus Lübeck	43	279
Charité Universitätsmedizin Berlin	47	NA
University Medical Center Göttingen	29	467
University Medical Center Knappschaftskrankenhaus Bochum	5	NA
University Hospital Cologne	5	410
University Hospital Tübingen	43	447
University Hospital Augsburg	177	1664
University Hospital Basel	35	326
University Medical Center Innsbruck	7	150
Total	442	4115

**Table 2:** Pre-existing conditions among patients with COVID-19, neurological symptoms and cranial imaging

Pre-existing conditions	*n* (%)
Diabetes mellitus	141 (31.9)
Cardiovascular (other than arterial hypertension)	147 (33.3)
Arterial hypertension	232 (52.5)
Cerebrovascular	64 (14.5)
Vascular other	20 (4.5)
Malignoma	74 (16.7)^*n*=440^
Renal	118 (26.7)

### Clinical information

Severity was mild in 124 patients (28%), moderate in 134 patients (30%), severe in 43 patients (10%) and critical in 141 (32%) patients.

Neurological symptoms persisted in 104 patients (23.5%), 189 patients (42.8) completely recovered, mortality was 27.8% (123 patients) during the hospital stay.

One hundred and seventeen patients (26.5%) were invasively ventilated during imaging. Median time of ventilation was 9 days at the time of imaging (range 0–48 days).

### Neurological symptoms

By far the most commonly reported neurological symptom (more than one symptom possible) was a decreased level of consciousness (*n* = 209/47.3%) and delirium (*n* = 162/36.7%, see Supplemental Materials).

### Time variables

Across all patients, 220 patients (49.8%) presented with “COVID first”, median time from COVID-19 symptoms onset to neurological symptoms onset was 7 days (range 0–58 days, *n* = 208, in the 12 remaining patients the interval was not recorded). 167 patients (37.8%) presented with “Neuro first”. The median time between the onset of neurological symptoms and the onset of COVID-19 symptoms was 2 days (range 0–37 days, *n* = 163, in the remaining 4 patients the interval was not recorded).

### Imaging

CT was performed in 406 patients, MRI in 91 patients and both CT and MRI in 55 patients. Contrast-enhanced CT was performed in 80 patients. Contrast-enhanced MRI was performed in 60 patients.

#### Acute ischemic stroke

**Fig. 2:**
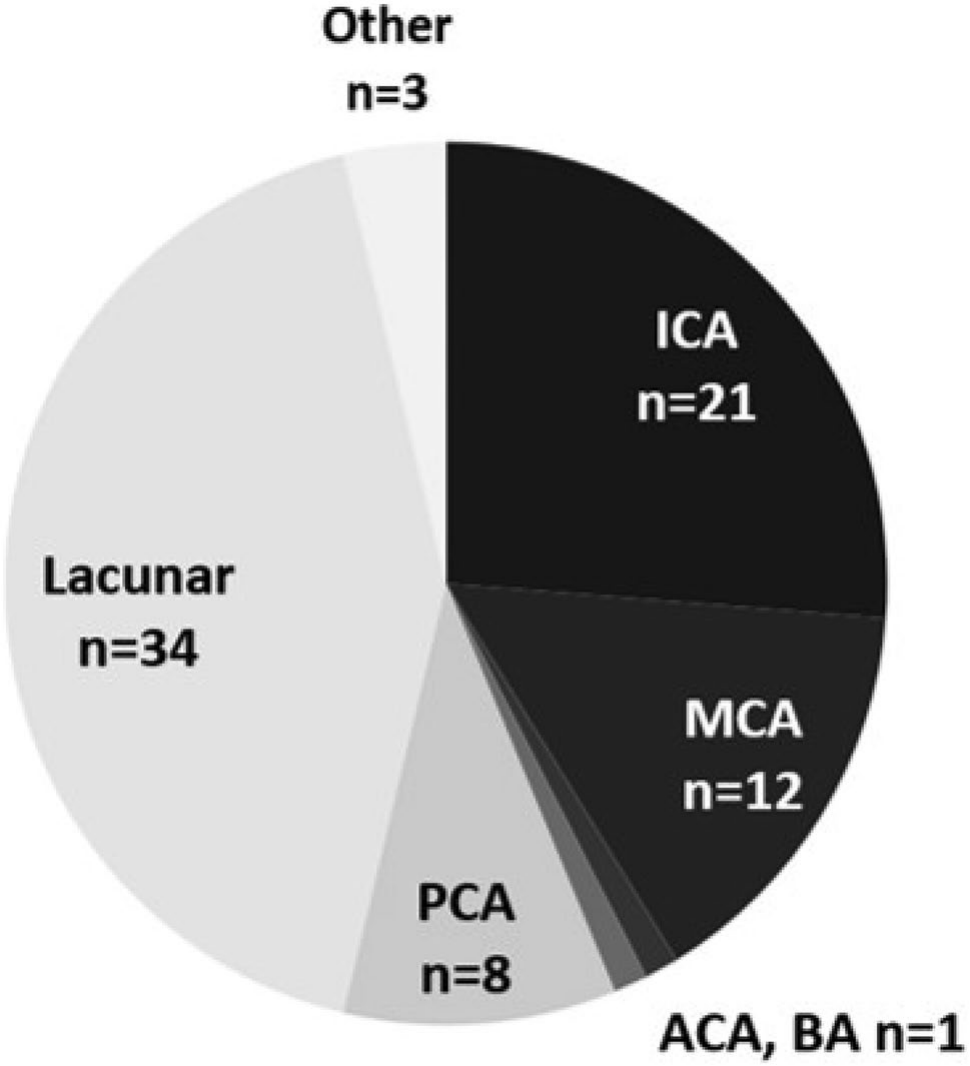
Vascular territory of acute cerebral ischemia (more than one territory possible). ICA (internal carotid artery), MCA (middle cerebral artery), anterior cerebral artery (ACA), basilar artery (BA), posterior cerebral artery (PCA)

AIS was detected in 70 (15.8%) patients (37 females (52.9%)). Patients with AIS were older than patients without (mean age 73.5 ± 13.1 years vs. 68.2 ± 16.4 years, *p* = 0.003, *t*-test). COVID-19 severity was mild *n* = 16 (22.9%), moderate *n* = 20 (28.6%), severe *n* = 6 (8.6%), critical *n* = 28 (40.0%). Based on all COVID-19 patients hospitalized during the study period, this corresponds to an incidence of 1.26% (52 events in 4115 patients in 8 centers). Details on the affected vascular territory can be found in Fig. 2. 90 patients (20.4%) had previous AIS. 31 patients presented with “COVID-19 first”, median time from COVID-19 symptoms onset to neurological symptoms onset was 12 days (range 0–35 days, *n* = 29, in the remaining 2 patients the interval was not recorded). 30 patients presented with “Neuro first”. The median time between the onset of neurological symptoms and the onset of COVID-19 symptoms was 1 day (range 0–15 days, *n* = 28, in the remaining 2 patients the interval was not recorded).

Mortality during the hospital stay was 30% (*n* = 21), neurological symptoms resolved in 24 patients (34.3%) and persisted in 23 patients (32.9%).

In patients with AIS, the median time to CT imaging was 0 days (range 0–15, *n* = 28 and 0–18 days, *n* = 25) in both “COVID first” and “Neuro first”, and 2 and 3 days, respectively, to MR imaging (range 1–3, *n* = 5 and 0–47 days, *n* = 8).

Neither invasive ventilation (*p* = 0.187, *χ*^2^-test) nor ECMO therapy (*p* = 0.379, *χ*^2^-test) was associated with AIS.

No association was found between the severity of COVID-19 and the occurrence of AIS (regular: OR = 1.07 (95% CI [0.52; 2.21]), *p* = 0.846; severe: OR = 0.79 [0.28; 2.22], *p* = 0.660; critical: OR = 1.52 [0.76; 3.03], *p* = 0.237).

Of the documented risk factors for and pre-existing conditions associated with AIS (arterial hypertension, diabetes mellitus, age, cerebrovascular, cardiovascular, vascular other) only arterial hypertension (*χ*^2^-test, *p* = 0.007, OR = 2.06) and cardiovascular preconditions (*χ*^2^-test, *p* = 0.0004, OR = 2.48) were associated with the presence of AIS. When fed into the model together with COVID-19 severity and age, the presence of any risk factors was associated with the presence of AIS (OR = 3.06 [1.31; 7.17], *p* = 0.005) while age was not (OR = 1.02 [1;1.04], *p* = 0.092).

#### Intracranial hemorrhages

**Fig. 3:**
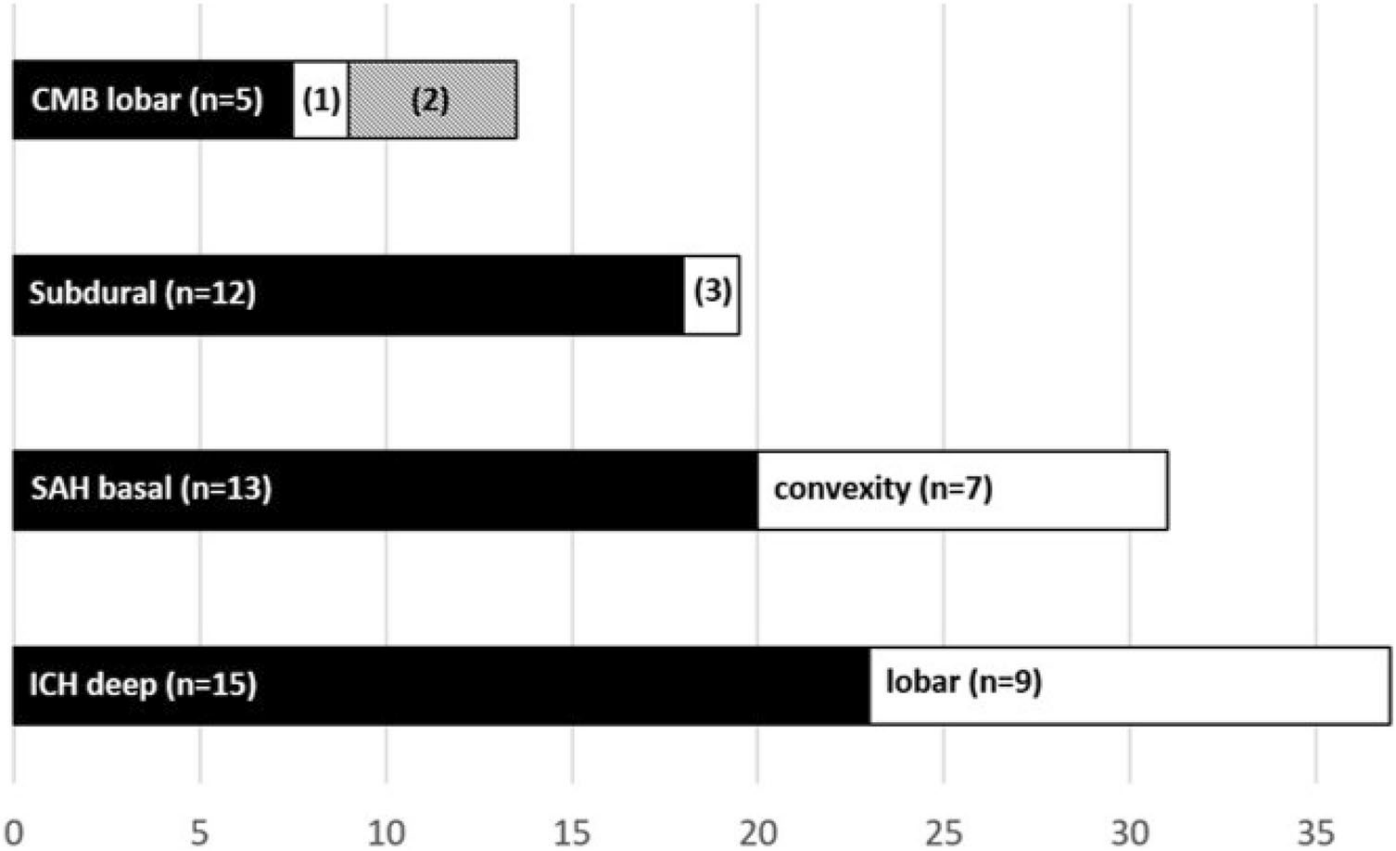
Location/type of hemorrhage (more than one location/type possible). 1: mixed, *n* = 1. 2: splenium of corpus callosum, *n* = 3. 3: epidural, *n* = 1

IH was detected in 48 (10.9%) patients (17 females (35.4%)). Patients with IH were younger than patients without IH (mean age 62.1 ± 15.9 years vs. 69.9 ± 15.9 years, *p* = 0.002, *t*-test). COVID-19 severity was mild *n* = 14 (29.2%), moderate *n* = 9 (18.8%), severe *n* = 1 (2.1%), critical *n* = 24 (50%). Based on all COVID-19 patients hospitalized during the study period, this corresponds to an incidence of 0.85% (35 events in 4115 patients in 8 centers). Details on location and type of hemorrhages can be found in Fig. 3.

Cerebral microbleeds (CMB) in mixed location (*n* = 1) and the corpus callosum (*n* = 3) were only detected in patients with critical COVID-19, altogether the incidence of CMB was 3.3% (*n* = 6) in severe and critically ill patients.

Ten IH in 8 patients (ICB *n* = 4, SAH *n* = 4, subdural *n* = 1 and epidural *n* = 1) were classified as traumatic, 2 subdural hematomas were rated as chronic, i.e., presumably pre-existing. After exclusion of these patients, the corrected incidence for IH in our study was 9.2%, and 0.7% for all COVID-19 patients hospitalized during the study period. The results of the other analysis did not change.

Mortality during the hospital stay was 37.5% (*n* = 18), symptoms resolved in 15 patients (22.0%) and persisted in 14 patients (29.2%).

Twenty patients presented with “COVID first” and the median time between the onset of COVID symptoms and the onset of neurological symptoms was 15 days (range 0–34 days, *n* = 17, in the remaining 3 patients the interval was not recorded). Seventeen patients presented with “Neuro first”. The median time between onset of neurological symptoms and the onset of COVID-19 symptoms was 3 days (range 0–11 days, *n* = 15, in the remaining 2 patients the interval was not recorded).

Median time to CT imaging in patients with IH was 0 days (range 0–15, *n* = 20 and 0–14 days, *n* = 15) for both “COVID first” and “Neuro first”, and 5 and 1 days, respectively, to MR imaging (range 1–15, *n* = 5 and 0–1 days, *n* = 3).

The use of antithrombotic drugs was not associated with the presence of IH (*p* = 0.066, *χ*^2^-test). ECMO therapy (30 vs. 9%, *p* < 0.001, Fisher’s exact test) and invasive ventilation (8 vs. 18%, *p* = 0.004, *χ*^2^-test) were associated with IH.

No association between the severity of COVID and the occurrence of IH could be detected (regular: OR = 0.6 [0.25; 1.46], *p* = 0.261; severe: OR = 0.23 [0.03; 1.82], *p* = 0.164; critical: OR = 1.74 [0.84; 3.59], *p* = 0.135).

The documented risk factors for and pre-existing conditions associated with IH (arterial hypertension, age) were not positively associated with the presence of IH (arterial hypertension OR = 0.60 [0.31; 1.17], *p* = 0.135; age OR = 0.98 [0.96;1], *p* = 0.029).

Detailed comparisons between the parameters of patients with AIS and IH are listed in Table 3.

**Table 3:** Comparison between AIS and IH in patients with COVID-19, neurological symptoms and cranial imaging

	AIS	IH	Difference* [95% CI]	*p* value
Total number, *n* (%)	70 (15.8)	48 (10.9)	0.050 [0.003; 0.097]	0.038
Age, mean ± SD, years	73.5 ± 13.1	62.1 ± 15.9	− 11.5 [− 17.0; − 5.9]	0.00008^#^
Female sex, *n* (%)	37 (52.9)	17 (35.4)	0.174 [− 0.022; 0.371]	0.093
COVID-19 severity, *n* (% per group)				
Mild	16 (22.9)	14 (29.2)	− 0.063 [− 0.243; 0.116]	0.577
Moderate	20 (28.6)	9 (18.8)	0.098 [− 0.072; 0.269]	0.318
Severe	6 (8.6)	1 (2.1)	0.065 [− 0.030; 0.159]	0.285
Critical	28 (40.0)	24 (50.0)	− 0.100 [− 0.300; 0.100]	0.376
Mortality, *n* (%)	21 (30.0)	18 (37.5)	− 0.075 [− 0.267; 0.117]	0.515
Neurological symptoms resolved, *n* (%)	24 (34.3)	15 (31.3)	0.030 [− 0.159; 0.220]	0.885
Neurological symptoms persisted, *n* (%)	23 (32.9)	14 (29.2)	0.037 [− 0.150; 0.224]	0.824
„COVID first“, *n* (%)	31 (44.3)	20 (41.7)	0.026 [− 0.173; 0.225]	0.926
Time from COVID symptoms-neurological symptoms, median (range), days	12 (0–35)^*n*=29^	15 (0–34)^*n*=17^	3 [− 3; 9]	0.284^##^
Time to CT, median (range), days	0 (0–15)^*n*=28^	0 (0–15)^*n*=20^	0 [− 0.00007; 1]	0.081^##^
Time to MRI, median (range), days	2 (1–3)^*n*=5^	5 (1–15)^*n*=5^	3 [− 1; 13]	0.109^##^
„Neuro first“, *n* (%)	30 (42.9)	17 (35.4)	0.074 [− 0.121; 0.270]	0.536
Time from neurological symptoms-COVID symptoms, median (range), days	1 (0–15)^*n*=28^	3 (0–11)^*n*=15^	1 [− 1; 3]	0.328^##^
Time to CT, median (range), days	0 (0–18)^*n*=25^	0 (0–14)^*n*=15^	0 [− 0.00003; 0.00006]	0.853^##^
Time to MRI, median (range), days	3 (0–47)^*n*=8^	1 (0–1)^*n*=3^	− 2 [− 6; 0.00001]	0.078^##^

Because eight patients had both AIS and IH, the test statistics are inflated and the *p* values therefore smaller. However, sensitivity analyses after exclusion of these eight patients show similar results (see Supplementary Table 3)

*Difference of proportions (test of equal proportions), difference of mean (*t*-test) or location shift (Wilcoxon rank sum test), #*t*-test, ##Wilcoxon rank sum test, all other tests: test of equal proportions

#### Other imaging findings

Other imaging findings are summarized in Table 4. 75% (*n* = 302) of the CTs and 52% (*n* = 40) of the MRIs did not show any findings potentially related to COVID-19.

**Table 4:** Other relevant imaging findings in patients with COVID-19, neurological symptoms and cranial imaging

Imaging findings	*n* (%)
Fluid collection in the paranasal sinus and/or mastoids	10 (2.3)
Hypoxic brain injury	8 (1.8)
Venous thrombosis	3 (0.68)
Hygroma	3
Probable postictal imaging abnormalities	3
Meningeal enhancement	2 (0.45)
Pathological olfactory nerve	2
Posterior reversible encephalopathy syndrome	2
Hemorrhagic pituitary infarct	1 (0.23)
Central pontine myelinolysis	1
Undetermined thalamic lesion	1
Undetermined juxtacortical lesion	1
Undetermined partial incomplete FLAIR suppression of CSF	1
Hyperdense pallidus globe	1

*FLAIR* fluid attenuated inversion recovery, *CSF* cerebrospinal fluid

### ECMO

33 patients had imaging after or during ECMO therapy. Patients were considerably younger than patients without ECMO therapy (60.7 ± 10.5 vs. 69.7 ± 16.2 years). Median time from ECMO to imaging was 3.5 days (range 0–42 days). Seven patients (21.2%) had AIS, and 10 patients (30.3%) suffered from an IH. Mortality was 45.5% (*n* = 15).

## Discussion

As the main results of our retrospective observational multicenter study, we can report the following:

1. We could not establish a correlation between severity of COVID-19 and AIS or IH.

Despite this being the imaging study with the largest target population [9, 10] on patients with COVID-19 and neurological symptoms in the acute stage to date, the number of patients included in the main analysis is still low. In contrast to comparable studies, we included hospitalized patients from a large catchment area [11] from three countries. Further, we included patients across the whole spectrum of COVID-19 severity [4, 12] and classified them according to standardized criteria [5].

Previous studies found a higher incidence of AIS in patients with more severe COVID-19 [13]. However, in contrast to these studies, we used a composite score following standardized criteria as a measure of COVID-19 severity. Notwithstanding this, there may be a correlation of individual surrogate parameters for COVID-19 severity (days on the intensive care unit, duration of ventilation, etc.) [14, 15] and the incidence of cerebrovascular events.

Although we saw a trend toward more events in severe and critical ill patients (50% of all events in 258 patients vs. 50% of all events in 184 patients), still 59 events occurred in mildly and moderately affected patients. These results demonstrate the susceptibility for neurological complications even in patients with mild and moderate symptoms of COVID-19.

2. IH was less frequent than AIS but still a highly prevalent finding, and patients with IH had a different clinical profile.

IH occurred less frequently than AIS, and in some cases could not be directly associated with COVID-19 but were rated as pre-existing or as sequela of trauma. However, the frequency of IH of app. 40% was much higher than expected when considering that app. 15% of strokes are hemorrhagic in a European population [16]. Patients with IH differed from patients with AIS in terms of ventilation, ECMO therapy and age. AIS was associated with the presence of typical risk factors. Further, in both patients’ groups (“COVID-19 first” and “Neuro first”), the time from onset of COVID-19 symptoms to onset of neurological symptoms and vice versa was slightly longer in patients with IH than in patients with AIS, although it did not reach statistical significance.

In patients with acute COVID-19 infection, the presumed mechanisms that may lead to AIS and IH largely overlap, namely endothelitis [17] with ensuing micro- and macrothrombosis (and potential subsequent vessel rupture) [18], downregulation of the angiotensin converting enzyme 2 receptor and resulting arterial hypertension [19], loss of vascular integrity [20] following general inflammation and cytokine storms. Given the aforementioned correlations and clinical observations, therapy-related secondary effects [21] may also play a pivotal role in this already complex scenario.

Patients with IH were considerably younger than patients with AIS. von Stillfried et al. reported the preferential use of ECMO therapy in younger patients with COVID-19 [22]. ECMO therapy is a risk factor for bleeding events and was also associated with the occurrence of IH in our study. Considering the possible influence of secondary iatrogenic effects due to more aggressive therapy in younger patients, this could explain at least a part of the differences but also the higher-than-expected frequency.

One pattern of IH deserving special attention is cerebral microbleeds (CMB). In our cohort, six of nine cases of this pattern occurred in severely ill patients. In the three remaining patients who had moderate disease course, the CMBs were in a superficial location as may be the case in pre-existing CAA [23]. It has been suggested that CMB occur in association with ECMO therapy or as a consequence of severe hypoxemia in patients with ARDS [24, 25]. In these patients an involvement of the splenium has been described, which was the case in 3 patients in our cohort.

Although the incidence of AIS in patients with acute COVID-19 is not as high as initially thought [26], it has been consistently reported to range from approximately 1% (France) [27] to 1.6% (USA) [3, 28] in all patients with COVID-19 (1.26% in this study) matching the frequency demonstrated here. Cho and co-workers [29] reported 1.5% in-hospital strokes (worldwide) as a complication, although this number was not broken down by AIS and IH.

Of note, lacunar stroke occurred more frequently than would be expected in a normal stroke cohort [30]. One could speculate that a generalized susceptibility to local thrombosis, as described in COVID-19, favors this stroke subtype. As in previous studies [8, 21, 22] of IH associated with COVID-19, a wide variety of bleeding patterns were observed without clear preponderance, suggesting potentially multiple or overlapping causative mechanisms. Although we cannot elucidate the mechanism of AIS or IH with this study, our results might serve to generate hypotheses for future studies.

3. A substantial proportion of patients presented with neurological symptoms first.

This finding emphasizes and justifies early triage and testing strategy for patients with neurological symptoms already recommended by several international panels [31, 32]. Although the time delay in the “Neuro first” group was relatively short (median 2 days) and infection may have been detected on admission for many patients, this still has implications for pre-admission testing, triage and staff protection in periods of high virus prevalence. We were not the first to describe this phenomenon. Nawabi et al. [8] described “neurological symptoms first” in two patients with IH without prior typical respiratory COVID-19 symptoms. However, we acknowledge that retrospective data acquisition and interstudy differences may have an impact on the dating of the exact symptom onset [33].

4. Imaging findings other than AIS and IH were rare.

Venous thrombosis was rarely reported in this study (3 cases, 0.68%) but is in line with the incidence across all COVID-19 patients [34] (in our study app. 72/100.000) but still much higher than the estimated incidence on the normal population (0.3–0.4/100.00) [35].

Of note, meningeal enhancement was rarely reported (*n* = 2), even in severe and critically ill patients. Helms and co-workers reported meningeal enhancement to be present in 8 of 13 patients in a cohort of severely ill patients in the early phase of the pandemic [4]. However, in this study and in following reports [9, 10], a dedicated FLAIR protocol rarely performed in routine imaging was used.

A pathology of the olfactory nerve was only reported in two patients. The olfactory nerve and olfactory system have been described as a potential entry route to the CNS [36, 37]. However, routine imaging is only rarely directed toward the olfactory nerve. Further, this finding is temporary, confined to the early stages of the disease [37].

Although this is the largest imaging-based study of acute stage patients presented to date, the number of cases included is too small to detect the true frequency of less common findings.

5. While a wide range of clinical symptoms was reported, AIS was associated with typical focal neurological deficits.

In line with previous reports, we found that delirium and reduced consciousness were the most common neurological symptoms [29, 38]. In our data, delirium was associated with AIS. However, many of the cases with these symptoms did not have a structural lesion, making it likely that they were encephalopathic symptoms not necessarily associated with acute findings on neuroimaging.

About 10% of all COVID-19 patients included in the study period showed CNS symptoms. This is less than the approximately 25% reported by Mao et al. in their first case series from Wuhan [2]. However, we included only patients who additionally had cranial imaging. Again, retrospective data acquisition and interstudy differences may have a pivotal impact on the frequency of neurological symptoms.

AIS was associated with typical focal neurological deficits whereas IH was not associated with specific neurological symptoms. Since we did not collect information on symptom severity, we cannot rule out that symptom severity had an influence on the quality of symptom detection. However, given the clinical experience that ischemic and hemorrhagic stroke are clinically undistinguishable, this seems unlikely.

The main weakness of our study is the lack of an adequate control group and the lack of longitudinal data. Due to the retrospective design, uniform imaging parameters and time-points, standardized neurological screening and laboratory or CSF testing were not possible. Although we assume that the findings are related to COVID-19, since they appeared in close temporal proximity to the infection, a causal relationship cannot be established with the acquired data. As a control group, one could imagine a cohort of patients (either historical or current with an infection of the respiratory tract other than COVID-19, e.g., influenza and the same inclusion and exclusion criteria as the cohort presented here. Other than that, neuropathological work-up of the central nervous system [39] even in some patients could establish a causal relationship between COVID-19 and the neurological symptoms and imaging findings presented here. Furthermore, as no previous imaging was available, it remains uncertain whether some findings were already present before the inclusion into the study. Caution has to be exerted when trying to draw generalized conclusions. A selection bias may be at hand since only data from university medical centers were included. Further, regional and temporal differences in virus mutations if characterized at all were not recorded and cannot be excluded.

Notable strengths of the study are the wide geographical range of patients included, the size of the sample cohort and the multilevel collection of parameters per patient (clinical data, imaging data, laboratory data). The inclusion of patients across the spectrum of COVID-19 severity allows for a comparison between mildly and moderately affected patients and those in severe and critical condition.

## Conclusion

This study adds to the growing body of work on neuroimaging, neurological and laboratory findings in patients with acute COVID-19. Using a composite severity scale, we found no association between AIS or IH and COVID-19 severity. IH was less common than AIS but more frequent than expected and patients with IH had a different clinical profile compared to patients with AIS. A considerable proportion of patients presented with neurological symptoms first, which should advocate stringent patient screening during periods of high virus prevalence. Laboratory parameters are of limited value as a screening tool for suspected pathological imaging findings.

## Supplementary Information

Below is the link to the electronic supplementary material.

Supplementary file1 (DOCX 39 KB)

## Data Availability

The data described in the manuscipt is available from the corresponding author upon reasonable request.
