# Supplementary material for: Patterns of acute ischemic stroke and intracranial hemorrhage in patients with COVID-19: Results of a retrospective multicenter neuroimaging-based study from three central European countries
Source: J Neurol. 2023 Feb 23;270(5):2349–59. doi: 10.1007/s00415-023-11608-2 (PMC9947908; doi:10.1007/s00415-023-11608-2)
Supplement: Supplementary file 1 — Supplementary file1 (DOCX 39 KB) [file 415_2023_11608_MOESM1_ESM.docx]

**SUPPLEMENTAL MATERIALS**

**MATERIAL AND METHODS**

COVID-19 severity scoring according to the “Handbook COVID-19 Prevention and Treatment”: It was classified as mild if patients were asymptomatic or had mild clinical symptoms and no pneumonia manifestation was seen on imaging. It was rated as moderate if patients had fever, respiratory symptoms or pneumonia on imaging. It was rated as severe if any of the following characteristics were present: respiration rate >30/min, oxygen saturation <93%, ratio of arterial oxygen partial pressure to fractional inspired oxygen (PaO_2_/FiO_2_) <= 300mmHg. It was rated as critical if any of the following was present: respiratory failure with mechanical ventilation, shock, other organ failure requiring intensive care.

**Blood laboratory parameters**

Blood samples were obtained at the time point closest to imaging but the exact time interval was not recorded. The results of the following blood laboratory parameters were dichotomized as being outside the normal range according to the local laboratory cut-off values (**Supplemental Table 1**): D-dimers, L-lactate dehydrogenase (LDH), creatinine, C-reactive protein (CRP), leukocyte and thrombocyte count.

**CSF parameters**

CSF was obtained at the time point closest to imaging but the exact time interval was not recorded. The results of the following CSF parameters were dichotomized as present or absent: Oligoclonal bands, elevated protein levels, and PCR positivity for SARS-CoV2.

**RESULTS**

**Neurological symptoms**

Focal-neurological symptoms were paresis (n=80/18.1%), speech impairment (n=70/15.8%), oculomotor dysfunction (n=32/7.2%), impaired sensory levels (n=27/6.1%), visual impairment (n=25/5.7%) and impaired smell (n=13/2.9%) and taste (n=12/2.7%). Non-focal neurological symptoms were headaches (n=57/12.9%), psychiatric disorders (n=50/11.3%), epileptic seizure (n=45/10.2%) and dizziness (n=29/6.6%). The following symptoms were only rarely reported: gait disorder, neglect, impaired swallowing (n=3, respectively) and hypokinesia, impaired hearing, transient global amnesia, and bladder incontinence (n=1, respectively).

Delirium (p=0.012, χ^2^-test), visual impairment (p=0.002, Fisher’s exact test), headaches (p=0.019, χ^2^-test), speech impairment (p<0.0001, χ^2^-test), impaired sensory levels (p=0.0002, Fisher’s exact test) and paresis (p<0.0001, χ^2^-test) were associated with acute cerebral ischemia. None of the neurological symptoms was associated with intracranial hemorrhages (p>0.05, χ^2^- or Fisher’s exact test as appropriate).

**CSF**

CSF was collected from 49 patients. Oligoclonal bands were present in 3 (10.7%) of 28 samples (COVID-19 severity mild n=2, critical n=1). Protein levels were elevated in 21 (42.9%) of 49 samples (COVID-19 severity mild n=8, moderate n=4, severe n=1, critical n=8). PCR for SARS-CoV2 was positive in 4 (23.5%) of 17 samples (COVID-19 severity mild n=2, moderate n=1, critical n=1). No association between CSF abnormalities and cerebral ischemia or intracranial hemorrhages could be demonstrated.

**Blood laboratory parameters**

Creatinine, leukocyte and thrombocyte count were available in all 442 patients and LDH and CRP were available in 441 out of 442 patients (99.7%). D-dimeres were available in only 325 out of 442 (74%) patients. D-dimers were pathologically elevated in 278 of 325 available samples (85.5%), LDH was elevated in 264 of 441 available samples (59.9%), creatinine was elevated in 189 of 442 available samples (42.8%), CRP was elevated in 370 of 441 available samples (83.7%), leucocytes were elevated in 123 of 442 samples (27.8%), and thrombocytes were reduced in 108 of 442 samples (24.4%). None of the parameters was associated with the presence of acute cerebral ischemia or intracranial hemorrhages. There is a strong association between COVID-19 severity and pathological blood laboratory parameters (**Supplemental Table 2**).

|  | **D-dimer** | **LDH** | **Creatinine** | **CRP** | **Leukocytes** | **Thrombocytes** |
| --- | --- | --- | --- | --- | --- | --- |
| University Medical Center Schleswig-Holstein, Campus Kiel | <0.79 µg/ml FEU | <250 U/l | 59-104 mg/l | <5 mg/dl | 3.9-10.2 n/nl | 150-370 n/nl |
| University Medical Center Schleswig-Holstein, Campus Lübeck | <0.79 µg/ml FEU | <250 U/l | 59-104 mg/l | <5 mg/dl | 3.9-10.2 n/nl | 150-370 n/nl |
| Charité Universitätsmedizin Berlin | 20-400 µg/l FEU | 135-250 U/l | 0.7-1.2 mg/dl | <5 mg/dl | 3.9-10.5 n/nl | 150-370 n/nl |
| University Medical Center Göttingen | <0.5 µg/ml FEU | 125-250 U/l | 0.7-1.2 mg/dl | <5 mg/dl | 4-11 n/nl | 150-350 n/nl |
| University Medical Center Knappschaftskrankenhaus Bochum | <0.5 µg/ml FEU | 135-224 U/l | 0.7-1.2 mg/dl | <0.5 mg/dl | 4-10 n/nl | 130-400 n/nl |
| University Hospital Cologne | <0.5 µg/ml FEU | <250 U/l | 0.5-1.1 mg/dl | <5 mg/dl | 4.4-11.3 n/nl | 150-400 n/nl |
| University Hospital Tübingen | <0.5 µg/ml FEU | <250 U/l | 0.6-1.1 mg/dl^m^  0.5-0.8 mg/dl^f^ | <0.5mg/dl | 3.8-10.3 n/nl | 150-450 n/nl |
| University Hospital Augsburg | <500 µg/l FEU | <250 U/l | 0.7-1.2 mg/dl^m^  0.5-0.9 mg/dl^f^ | <0.5 mg/dl | 3-10 n/nl | 140-440 n/nl |
| University Hospital Basel | 0.19-0.5 µg/ml FEU | 135-225 U/l | 49-97 mg/l | <10 mg/d | 3.5-10 n/nl | 150-450 n/nl |
| University Medical Center Innsbruck | <500 µg/l FEU | 100-500 U/l | 0.67-1.17 mg/dl | <0.5 mg/dl | 4-10 n/nl | 150-380 n/nl |
|  |  |  |  |  |  |  |

**Supplemental Table 1**: Pathological blood parameters, n (%), and association (β: logistic regression coefficient, OR: odds ratio) with COVID severity scores.

|  | **Total (n=442)** | **Mild (n=124)** | **Moderate (n=124)** | **Severe (n=43)** | **Critical (n=119)** |
| --- | --- | --- | --- | --- | --- |
| **D-dimer, n (%)** | 278 (86)^n=325^ | 57 (71)^n=80^ | 78 (82)^n=95^ | 27 (87)^n=31^ | 116 (97)^n=119^ |
|  |  |  | β=0.62, p=0.091, OR=1.85 [0.91;3.78] | β=1.00, p=0.089, OR=2.72 [0.86; 8.66] | **β=2.75, p<0.001**, **OR=15.60 [4.50; 54.14]** |
| **LDH, n (%)** | 264 (59.7)^n=441^ | 55 (45)^n=123^ | 82 (61.2) | 28 (65.1) | 99 (70.2) |
|  |  |  | **β=0.67, p=0.008**, **OR=1.95 [1.19; 3.20]** | **β=0.84, p=0.020, OR=2.31 [1.12; 4.75]** | **β=1.07, p<0.001**, **OR=2.91 [1.76; 4.84]** |
| **Creatinine, n (%)** | 189 (42.8) | 42 (33.9) | 48 (35.8) | 29 (67.4) | 70 (49.6) |
|  |  |  | β=0.09, p=0.743, OR=1.09 [0.65; 1.82] | **β=1.40, p<0.001, OR=4.04 [1.93; 8.46]** | **β=0.65, p=0.010**, **OR=1.92 [1.17; 3.16]** |
| **CRP, n (%)** | 370 (83.7)^n=441^ | 84 (67.7) | 111 (82.8)^n=123^ | 41 (95) | 134 (95) |
|  |  |  | **β=0.88, p=0.004, OR=2.40 [1.33; 4.34]** | **β=2.28, p=0.002**, **OR=9.76 [2.25; 42.38]** | **β=2.21,** **p<0.001, OR=9.12 [3.90; 21.29]** |
| **Leucocytes, n (%)** | 123 (27.8) | 24 (19.4) | 22 (16.4) | 11 (25.6) | 66 (46.8) |
|  |  |  | β=-0.20, p=0.538, OR=0.82 [0.43; 1.55] | β=0.36, p=0.389, OR=1.43 [0.63; 3.24] | **β=1.30,** **p<0.001**, **OR=3.67 [2.11; 6.39]** |
| **Thrombocytes, n (%)** | 108 (24.4) | 27 (21.8) | 38 (28.4) | 19 (44.2) | 24 (17) |
|  |  |  | β=0.35, p=0.225, OR=1.42 [0.81; 2.51] | **β=1.05, p=0.005**, **OR=2.84 [1.36; 5.95]** | β=-0.31, p=0.328, OR=0.74 [0.40; 1.36] |

**Supplementaal Table 2**: Normal range of laboratory parameters.

FEU: fibrinogen equivalent units

|  | | **AIS** | **IH** | **Difference^*^ [95% CI]** | **p-value** |  |
| --- | --- | --- | --- | --- | --- | --- |
| Total number, n (%) | | 62 (14.3) | 40 (9.2) | 0.05  [0.006; 0.096] | 0.027 |  |
| Age, mean ± SD, years | | 74.3 ± 12.5 | 60.9 ± 15.7 | -13.4  [-19.2; -7.5] | 0.00002^#^ |  |
| Female sex, n (%) | | 35 (56.5) | 15 (37.5) | 0.19  [-0.025; 0.404] | 0.096 |  |
| COVID-19 severity, n (% per group) | mild | 15 (24.2) | 13 (32.5) | -0.08  [-0.284; 0.118] | 0.490 | |
|  | regular | 18 (29.0) | 7 (17.5) | 0.11  [-0.068; 0.299] | 0.277 | |
|  | severe | 6 (9.7) | 1 (2.5) | 0.07  [-0.037; 0.180] | 0.318 | |
|  | critical | 23 (37.1) | 19 (47.5) | -0.104  [-0.321; 0.113] | 0.403 | |
| Mortality, n (%) | | 19 (30.6) | 16 (40.0) | -0.09  [-0.304; 0.117] | 0.448 |  |
| Neurological symptoms resolved, n (%) | | 21 (33.9) | 12 (30.0) | 0.04  [-0.166; 0.244] | 0.848 |  |
| Neurological symptoms persisted, n (%) | | 20 (32.3) | 11 (27.5) | 0.05  [-0.154; 0.249] | 0.772 |  |
| „COVID first“, n (%) | | 28 (45.2) | 17 (42.5) | 0.03  [-0.191; 0.244] | 0.952 |  |
| Time from COVID symptoms-neurological symptoms, median (range), days | | 10 (0-35)^n=26^ | 12.5 (0-33)^n=14^ | 3  [-4; 10] | 0.300^##^ |  |
| Time to CT, median (range), days | | 0 (0-15)^n=25^ | 1 (0-15)^n=17^ | 0.0000004  [-0.000007; 1] | 0.065^##^ |  |
| Time to MRI, median (range), days | | 2 (1-3)^n=4^ | 5 (3-15)^n=4^ | 3  [0.00005; 14] | 0.040^##^ |  |
| „Neuro first“, n (%) | | 27 (43.5) | 14 (35.0) | 0.09  [-0.128; 0.299] | 0.514 |  |
| Time from neurological symptoms-COVID symptoms, median (range), days | | 1 (0-15)^n=26^ | 3 (0-11)^n=13^ | 1  [-1; 3] | 0.352^##^ |  |
| Time to CT, median (range), days | | 0 (0-18)^n=23^ | 0 (0-11)^n=13^ | 0  [-0.00002; 0.000004] | 1^##^ |  |
| Time to MRI, median (range), days | | 4 (0-47)^n=7^ | 0.5 (0-1)^n=2^ | -4  [-47; 0.00005] | 0.083^##^ |  |

**Supplementary Table 3**: Comparison between AIS and IH in patients with COVID-19, neurological symptoms and cranial imaging after exclusion of eight patients with both AIS and IH.

*: Difference of proportions (test of equal proportions), difference of mean (t-test) or location shift (Wilcoxon rank sum test), #: t-test, ##: Wilcoxon rank sum test, all other tests: test of equal proportion

**DISCUSSION**

1. Blood laboratory and CSF parameters were not associated with pathological findings on acute neuroimaging.

Earlier reports saw it differently, but they were clearly hampered by very small case numbers^1^. However, laboratory parameters were associated with severity of COVID-19. This finding is in line with previous larger studies. Especially, C-reactive protein as an inflammatory marker and D-dimers as an indicator of coagulation activation, which also occurs in inflammation, are regularly elevated in patients with severe and critical COVID-19 and have been identified as predictors of poor outcome and mortality (for reviews see^2,3^). Thus, the value of these parameters as a screening tool for pathological findings in neuroimaging is limited. Notably, in line with previous reports^4^, SARS-CoV2 was rarely found in CSF and was not associated with neuroimaging findings, supporting the notion that most findings are the results of secondary inflammatory events leading to injury of the CNS. However, there is strong selection bias as only a small fraction of patients had a CSF sample available. Further, as no information on the time interval between the onset of neurological symptoms and the date of the blood or CSF sample is available, this introduces an additional unknown bias.

**REFERENCES**

1. Katz JM, Libman RB, Wang JJ, et al. COVID-19 Severity and Stroke: Correlation of Imaging and Laboratory Markers. *American Journal of Neuroradiology* 2021; 42: 257–261.

2. Rostami M, Mansouritorghabeh H. D-dimer level in COVID-19 infection: a systematic review. *Expert Rev Hematol* 2020; 13: 1265–1275.

3. Luan Y, Yin C, Yao Y. Update Advances on C-Reactive Protein in COVID-19 and Other Viral Infections. *Front Immunol*; 12. Epub ahead of print 10 August 2021. DOI: 10.3389/fimmu.2021.720363.

4. Jarius S, Pache F, Körtvelyessy P, et al. Cerebrospinal fluid findings in COVID-19: a multicenter study of 150 lumbar punctures in 127 patients. *J Neuroinflammation* 2022; 19: 19.
